# Slight reduction in SARS-CoV-2 exposure viral load due to masking results in a significant reduction in transmission with widespread implementation

**DOI:** 10.1038/s41598-021-91338-5

**Published:** 2021-06-04

**Authors:** Ashish Goyal, Daniel B. Reeves, Niket Thakkar, Mike Famulare, E. Fabián Cardozo-Ojeda, Bryan T. Mayer, Joshua T. Schiffer

**Affiliations:** 1 Vaccine and Infectious Diseases Division, Fred Hutchinson Cancer Research Center, Seattle, USA; 2 Institute for Disease Modeling, Global Health Division, Bill and Melinda Gates Foundation, Seattle, USA; 3 Department of Medicine, University of Washington, Seattle, USA; 4 Clinical Research Division, Fred Hutchinson Cancer Research Center, Seattle, USA

**Keywords:** Computational models, Viral infection

## Abstract

Masks are a vital tool for limiting SARS-CoV-2 spread in the population. Here we utilize a mathematical model to assess the impact of masking on transmission within individual transmission pairs and at the population level. Our model quantitatively links mask efficacy to reductions in viral load and subsequent transmission risk. Our results reinforce that the use of masks by both a potential transmitter and exposed person substantially reduces the probability of successful transmission, even if masks only lower exposure viral load by ~ 50%. Slight increases in mask adherence and/or efficacy above current levels would reduce the effective reproductive number (R_e_) substantially below 1, particularly if implemented comprehensively in potential super-spreader environments. Our model predicts that moderately efficacious masks will also lower exposure viral load tenfold among people who get infected despite masking, potentially limiting infection severity. Because peak viral load tends to occur pre-symptomatically, we also identify that antiviral therapy targeting symptomatic individuals is unlikely to impact transmission risk. Instead, antiviral therapy would only lower R_e_ if dosed as post-exposure prophylaxis and if given to ~ 50% of newly infected people within 3 days of an exposure. These results highlight the primacy of masking relative to other biomedical interventions under consideration for limiting the extent of the COVID-19 pandemic prior to widespread implementation of a vaccine. To confirm this prediction, we used a regression model of King County, Washington data and simulated the counterfactual scenario without mask wearing to estimate that in the absence of additional interventions, mask wearing decreased R_e_ from 1.3–1.5 to ~ 1.0 between June and September 2020.

## Introduction

Masks are a barrier method to prevent the spread of respiratory viral infections. A mask essentially serves as a filter that prevents passage of some portion of viruses from the airway of the transmitter to the airway of exposed contacts. Mask efficacy is therefore mediated by a reduction in exposure viral load which we define as the virus that reaches the airway of the exposed contact after the filtration from masks used by transmitter and/or exposed contact^1,2^. If a potential transmitter as well as an exposed contact are masked, then this filtering process occurs twice potentially amplifying protection.

Mask efficacy is inferior to that of other, less permeable barrier methods to prevent infections such as condoms^1,3,4^. The most commonly used cloth and hospital masks do not provide a perfect facial seal and mask fabric does not block emission or inhalation of all aerosolized viral particles^5,6^. N95 masks may bypass these shortcomings but are in short supply and difficult to wear for long periods of time^7,8^. As a result of these imperfections, recommendations for mask use have varied over the course of the COVID-19 pandemic. Nevertheless, widespread mask use is recognized as a critical component of any viable public health strategy against COVID-19^9–11^. Recent models demonstrate that slight increases in mask utilization could be the single most important factor that prevents exponential growth in incident cases^12–14^.

Quantifying mask efficacy in real-world settings remains challenging. Elegant experimental work demonstrated the efficacy of masks in animal models^15^. Many studies have been performed in hospital settings where mask compliance is uniform and other complementary infection prevention methods are more commonly employed than in other public gathering or work locations^16,17^. To the best of our knowledge, no study has captured the impact of masking on the likelihood of super-spreader events, with specific consideration of intermittent compliance.

Here we develop a mathematical model capturing viral load-mediated effects of mask use on transmission probability within transmission pairs and at the population level. We use this approach to estimate the efficacy of masks in real world settings, and to characterize effects on super-spreader events as well as exposure viral loads of those who get infected despite masking. We also validated our conclusion about the outsized impact of mask wearing using epidemiological data from Washington state following a mask mandate—by estimating the transmission reductions due to mask wearing and simulating a counterfactual scenario without masks. Finally, we compare the preventative impact of masking to the use of antiviral therapies given early during symptomatic infection, or when used as post-exposure prophylaxis (PEP).

## Results

### Baseline mathematical model of SARS-CoV-2 viral load dependent transmission

To determine the impact of masks on epidemics, we employed two of our previously developed modeling frameworks including (i) a within-host viral dynamics model whose parameters are informed by the fitting of 25 untreated SARS-CoV-2 infected individuals^18^, and (ii) a multi-scale model which links viral load shedding at the individual level with population level epidemic spread^19^. The latter model is built upon the assumptions that each transmitter has a specific number of exposure contacts per day and that each exposure contact has a certain probability of successful transmission based on the transmitter viral load (i.e., the measured SARS-CoV-2 viral loads in the transmitter via nasal swabs). This probability is based on a transmission dose (TD) response curve, which we characterize by fitting the model to mean R0 as well as frequency histograms describing heterogeneity of individual R0^19^, or the number of secondary infections attributed to each infected person obtained from the contact tracing data of 391 SARS-CoV-2 cases in Shenzhen, China among other locations^20–24^. The model was simultaneously fit to mean serial interval, the time from the onset of symptoms in the transmitter to symptom onset in the secondarily infected person, and distributions of individual serial intervals obtained from the data on 468 COVID-19 transmission events reported in mainland China^25^. Of note, the individual R0 distribution for SARS-CoV-2 transmission is highly over-dispersed, meaning that most infected people do not spread infection while a minority infect a large number of people. The distribution of serial interval of SARS-CoV-2 is inclusive of negative values, which is suggestive of the occasional onset of symptoms in the secondarily infected person before the onset of symptoms in the transmitter. The overall qualitative conclusions of this model were that the period of contagiousness for SARS-CoV-2 is quite short, typically less than a day, and that super-spreader events are largely attributable to high variability in the number of exposure contacts per day among infected people^19^.

### Predicted impact of transmitter or exposure contact masking on transmission probability within transmission pairs

**Figure 1:**
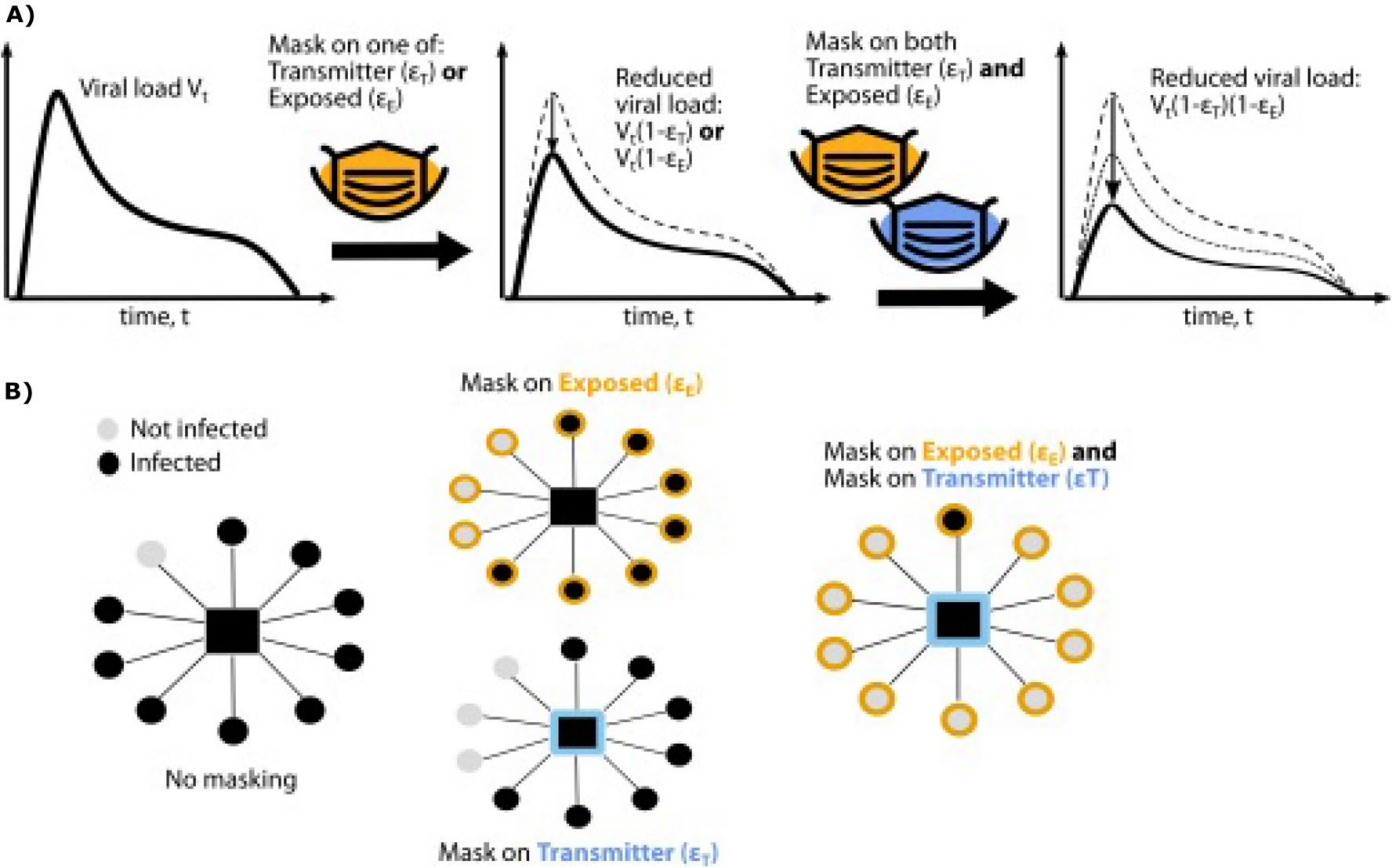
Schematic of mask impact on SARS-CoV-2 exposure viral load. (**A**) The viral load emitted by a potential transmitter (VT) can be filtered, resulting in lower exposure viral loads due to a single mask worn by a transmitter or exposed individual with efficacy *ε*_T_ or *ε*_E_ respectively. Dual masking lowers exposure viral load further by filtering virus twice. (**B**) Dual masking may prevent super-spreader events to a greater extent than a masked transmitter or masked exposed individual.

We added masking to this model by assuming that a mask decreases the exposure viral load in a transmission pair by a value that we refer to as the combination mask efficacy (*ε*_*C*_). This efficacy represents the proportion of viruses filtered by masks worn by both the transmitter and exposed person. If the transmitter is wearing a mask with efficacy *ε*_*T*_ and the exposed person is wearing a mask with efficacy *ε*_*E*_, then the exposure viral load *V*_*E*_ can be related to the transmitter viral load *V*_*T*_ by: *V*_*E*_ = *V*_*T*_ (1 − *ε*_T_)(1 − *ε*_E_). The combination mask efficacy is then *ε*_*C*_ = 1 − (1 *− ε*_T_)(1 *− ε*_E_), which takes on a value of zero when both parties are not wearing a mask or wearing masks that are totally ineffective (Fig. 1).

As in our prior model, the exposure viral load impacts contagiousness, which is the probability that virus is passaged to the exposed person’s airway, as well as infectiousness, the probability of cellular infection given the presence of virus in the airway. Each of these properties is associated with a dose response curve (contagiousness dose (CD) response curve and infectiousness dose (ID) response curve), the product of which is the TD response curve.

**Figure 2:**
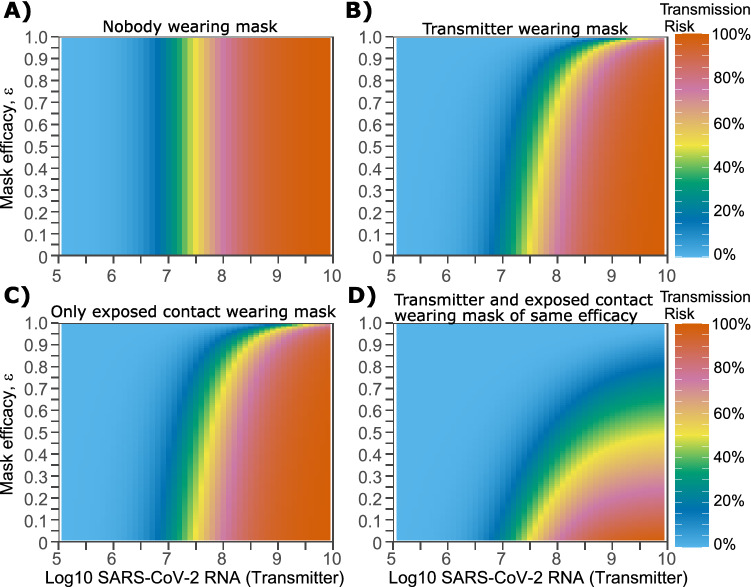
Impact of masking the transmitter alone, the exposed contact alone or both members of the transmission pair, on transmission risk given a single exposure contact. (**A**–**D**) Each panel is based on simulations of 1000 transmission pairs. (**A**) No masking, (**B**) Transmitter is masked, (**C**) Exposed contact is masked, (**D**) Both members are masked.

In this context, we first establish a baseline probability of transmission given no use of masks on both sides of a potential transmission pair (Fig. 2A). The absolute & relative reductions in transmission probability with more effective masks vary as *V*_*T*_ increases. At lower viral loads (< 10^7.5^ viral RNA copies), a moderate to highly effective mask worn by either a transmitter (0.9 > *ε*_T_ ≥ 0.5) or an exposed contact (0.9 > *ε*_E_ ≥ 0.5) is sufficient to partially lower the absolute probability of transmission (Fig. 2B, C, Supplementary Fig. 1A). The relative reduction in transmission probability increases linearly with increasing mask efficacy at 10^7^ viral RNA copies with an increasingly concave, curvilinear relationship at higher viral loads (Supplementary Fig. 1B).

At higher transmitter viral load (10^7.5^–10^9^ viral RNA copies), a moderate to highly effective mask worn by either a transmitter (0.9 > *ε*_T_ ≥ 0.5) or an exposed contact (0.9 > *ε*_E_ ≥ 0.5) insignificantly lowers the absolute probability of transmission (Fig. 2B, C, Supplementary Fig. 1A). At high viral loads, the relative reduction in transmission probability increases dramatically with extremely effective masks of efficacy *ε*_x_ ≥ 0.9, when the mask is worn by either a transmitter or an exposed contact (Supplementary Fig. 1B).

### Predicted impact of dual masking on transmission probability within transmission pairs

If both the transmitter and exposed person wear masks (dual masking), then lower mask efficacies are sufficient to significantly lower transmission risk at a wider range of exposure viral loads (Fig. 2D). At viral loads < 10^8^ viral RNA copies, masks worn by both transmitter and exposed contact of more than moderate efficacy (*ε*_T_ ≥ 0.5, *ε*_T_ ≥ 0.5 resulting in *ε*_C_ ≥ 0.75), is sufficient to partially lower the absolute probability of transmission (Fig. 2D, Supplementary Figs. 1C, 2A–C). The relative reduction in transmission probability according to mask efficacy increases more rapidly with dual (Supplementary Fig. 1D) compared to single (Supplementary Fig. 1B) masking. If both transmitter and exposed contacts wear masks with *ε*_T_ = 0.9 and *ε*_*E*_ = 0.9 (*ε*_*C*_ = 0.99), then transmission probability is reduced to < 5% for viral loads < 10^8.5^ viral RNA copies and to ~ 20% for transmitter viral load of 10^9^ viral RNA copies (Fig. 2D, Supplementary Figs. 1C, and 2D).

### Predicted impact of transmitter and exposure contact masking on effective reproduction number (R_e_) at different levels of implementation

**Figure 3:**
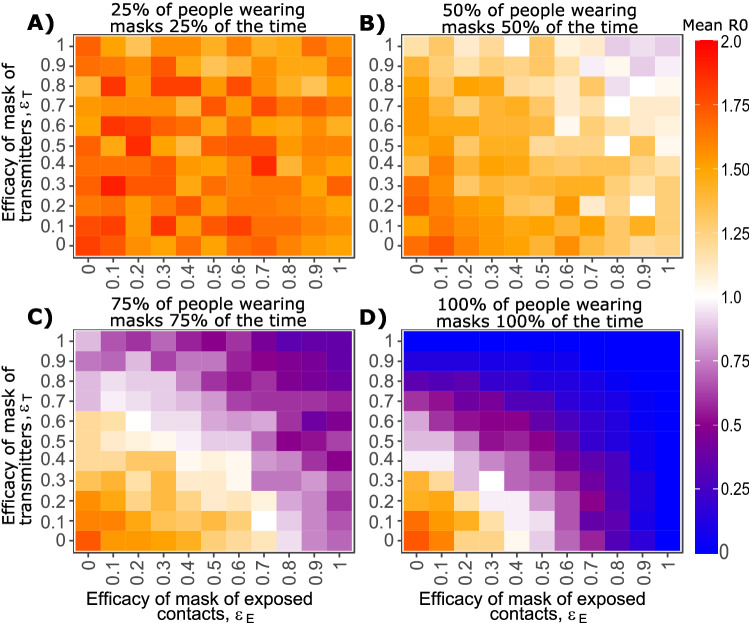
Effect of mask utilization and efficacy on mean SARS-CoV-2 effective reproductive number. Heat maps (color denotes basic reproductive number) with varying efficacy for both exposed and transmitters. Each panel includes simulations of 3000 transmitters with varying daily exposure contacts and (**A**) low (**B**) moderate (**C**) high, and (**D**) perfect mask usage. Specifics of mask use are noted in panel titles.

We next explore the impact of general masking adherence rates on population level metrics of infection by simulating 3000 potential transmitters assuming heterogeneity in viral load trajectories and exposure contact networks among individuals. Reduction in the effective reproductive number (R_e_) depends on both the mask efficacy levels (*ε*_T_ and *ε*_E_) and the level of adherence to masking (Fig. 3). If we assume 25% of people wear masks 25% of the time, where time refers to 24 h in a full day, then most variability in results occur due to the stochastic nature of the model (Fig. 3A). If we assume that 50% of people wear masks 50% of the time, then the use of masks with high efficacy of ~ 0.9 results in a drop of R_e_ from ~ 1.8 to ~ 1.0 (Fig. 3B). With 75% of people wearing masks 75% of the time, a mask efficacy of ~ 0.5 allows for a reduction of R_e_ from ~ 1.8 to ~ 1.0 (Fig. 3C). With 100% of people wearing masks 100% of the time, then a mask efficacy of ~ 0.3 is sufficient to achieve R_e_ ~ 1.0, and efficacy of 0.5 in both transmitter and exposed contacts lowers R_e_ to less than 0.6 (Fig. 3D).

Estimates of daily mask use (which is equivalent to the product of “percent of people wearing masks” and “percent of time wearing masks” in our simulations) in October, 2020 varied between states in the United States between 65 and 95%^26^, though local surveys suggest that masking rates in high risk private environments are lower (~ 40%) relative to in public settings^27^. R_e_ has varied between 0.8 and 1.2^28^. These results suggest that panel Fig. 3C, D is likely the closest to recent U.S. epidemic conditions and that ε_T_ and ε_E_ likely fall roughly between 0.3 and 0.6 in a real-world setting, if mask efficacy is equivalent between transmitter and exposed contacts. While a combined efficacy *ε*_C_ of 0.5–0.85 can be roughly estimated from the model, the possibility of superior efficacy of masks in transmitters versus exposed, or vice versa, cannot be excluded.

### Proportions of infections attributable to masked and unmasked transmission pairs

**Figure 4:**
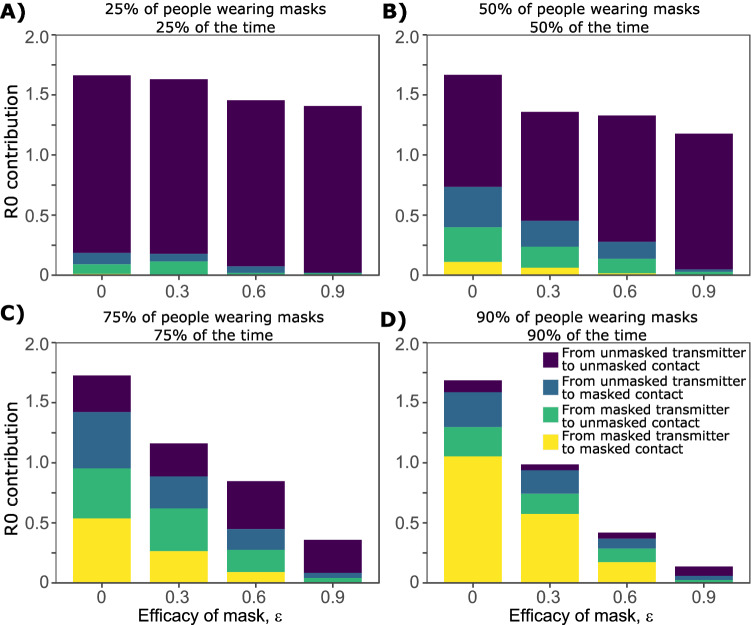
Effect of mask utilization and efficacy on proportion of masked transmissions contributing to total R_0_. Each panel includes simulations of 3000 transmitters with varying daily exposure contacts and (**A**) low (**B**) moderate (**C**) high, and (**D**) near-perfect mask usage. Specifics of mask use are noted in panel titles.

We next project the proportion of transmission events attributable to different masking profiles among transmission pairs assuming equally effective mask (*ε*_*x*_) used by both transmitters and exposed contacts. In circumstances with low mask utilization (25% of people wearing masks 25% of the time), nearly all transmissions occur from an unmasked person to an unmasked person (Fig. 4A). A similar trend is noted for moderate mask utilization (50%), particularly as mask efficacy increases (Fig. 4B).

For high (75%) and extremely high (90%) mask utilization scenarios, if mask efficacy is moderate (*ε*_*x*_ ~ 0.6) as is currently believed, then a higher proportion of transmissions occur to or from a person wearing a mask, despite the fact that the total number of transmissions dramatically decreases (Fig. 4C, D).

We project that at recent epidemic conditions in the United States, including ~ 50% effective mask use (similar to 75% of people wearing masks 75% of the time) and mask efficacy of 0.3–0.6, that fewer than half of ongoing transmissions occur within unmasked pairs and that transmission between masked transmitters or masked exposed contacts likely contributes significantly to R_e_ (Fig. 4C).

### Predicted impact of transmitter and exposure contact masking on super-spreader events

We next identified that increased mask compliance and efficacy dramatically decreases the proportion of infected people who successfully transmit to another person (Supplementary Fig. 3A–D). If 50% of people were to wear 50% effective masks half of the time, then the likelihood of an individual transmitting decreases from 30 to 20% (Supplementary Fig. 3B). If mask compliance is increased to 75% of people 75% of the time, then the likelihood of a person transmitting decreases to ~ 15% (Supplementary Fig. 3C).

When masking is applied homogeneously across the population, the proportion of infectors (transmitters who infect at least one person) who pass the infection to 5 or more people decreases, as mask utilization and efficacy increase (Supplementary Fig. 3E–H). Increased mask utilization and increased mask efficacy leads to an even reduction of all types of transmission events, including transmissions to small numbers (1–3) of people, or super-spreader events to > 5, > 10, > 20 or > 50 people (Supplementary Fig. 4A–D). Improvements in mask efficacy have a larger impact as utilization of mask use increases, including against super-spreader events (Supplementary Fig. 4C, D). Under all simulations, super-spreader events with transmission to > 5 people persist and make a nearly equivalent contribution proportionally to overall R_e_, though their absolute impact is considerably lessened with higher mask compliance and efficacy.

Our results suggest that with current levels of masking in the United States (Supplementary Fig. 4C, *ε*_*x*_ = 0.3–0.6), most of the contribution to R_e_ still comes from super-spreader events involving > 5 secondary infections. We therefore simulated masking applied to 100% of people with > 10 exposure contacts per day (Supplementary Fig. 5A–D) and found that even modest uptake of moderately effective masks (*ε* ~ 0.5) in the remainder of the population appeared to maintain R_e_ < 1.

### Predicted impact of transmitter and exposure contact masking on viral inoculum at time of infection

**Figure 5:**
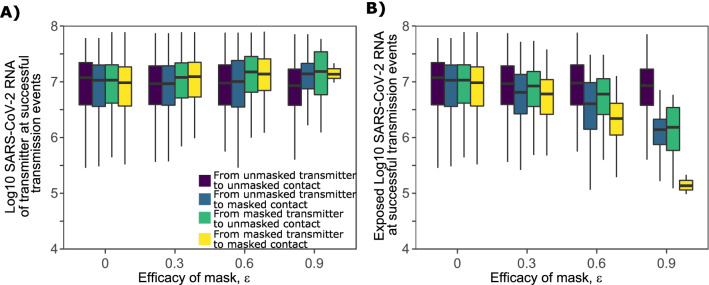
Effect of mask efficacy on exposure viral load during successful transmissions. Viral loads of the transmitter plotted against mask efficacy. Colors indicate masking conditions (see legend). Each boxplot in each panel is based on simulations of 3000 transmitters with varying daily exposure contacts. (**A**) Viral load of transmitters and (**B**) viral load exposure in infected individuals.

Another theoretical benefit of masks is reduction in exposure viral load which in animal models of SARS-CoV-1 and MERS, leads to less severe infection^29–31^. Simulating under the assumption that 75% of people wear masks 75% of the time (i.e., a situation representing recent levels of masking in the United States), we identified that transmitter viral load required to generate secondary infections increases slightly with higher implementation of more efficacious masks, particularly with dual masking of transmitters and exposed contacts (Fig. 5A). Exposure viral load at the time of a successful transmission decreased according to efficacy of mask, particularly if both transmitters and exposed contacts are masked (Fig. 5B). With dual masking in place with efficacies of 0.6, exposure viral load decreased by ~ 1 log (Fig. 5B). With dual masking in place with efficacies of 0.9, exposure viral load decreased by ~ 2 logs (Fig. 5B).

### Model validation using data from King County, Washington

**Figure 6:**
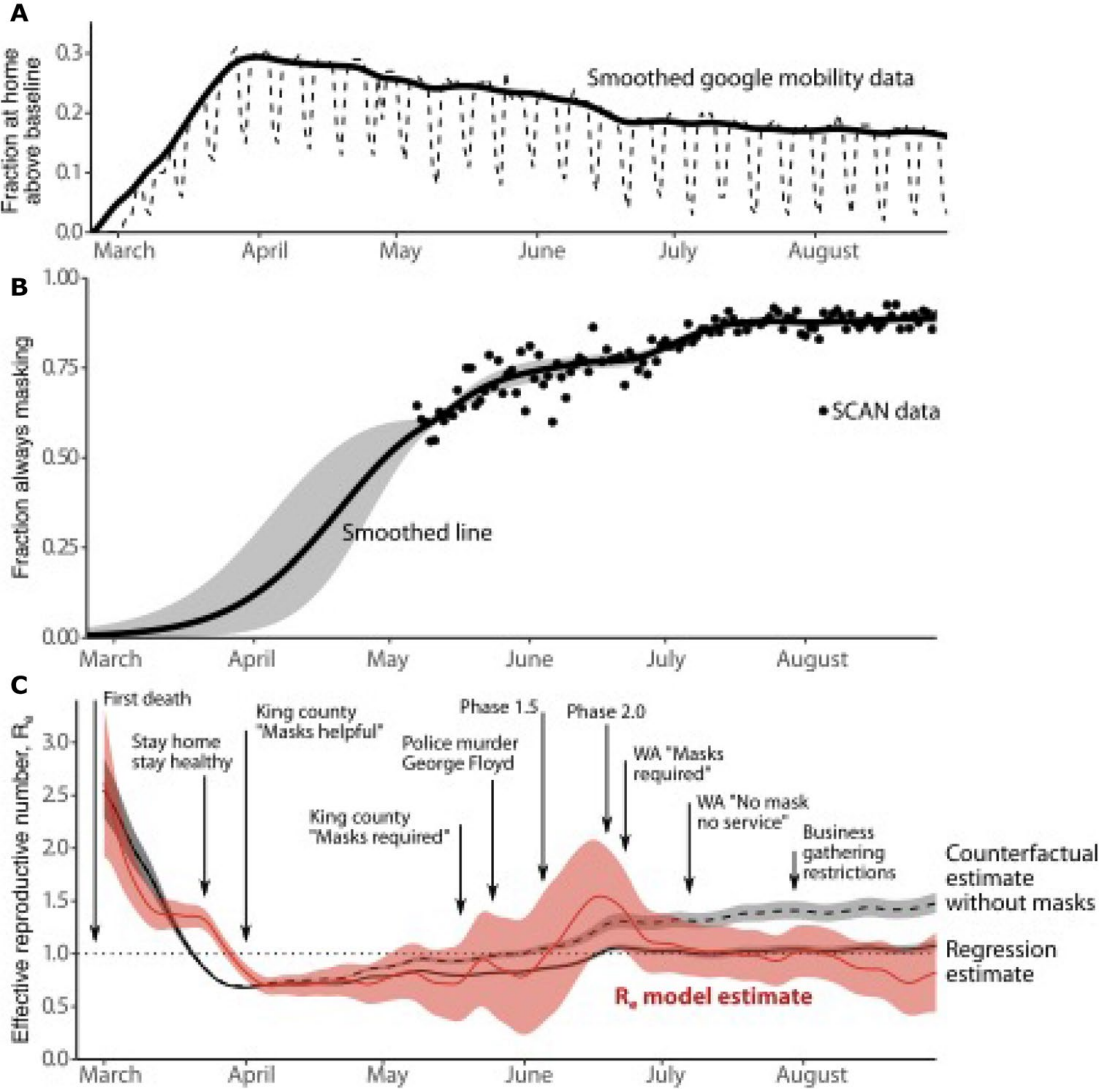
Empirical estimated impact of mask usage in King County, Washington. Regression model estimates of reduction in effective reproductive number (R_e_) explained by human mobility and mask wearing. (**A**) Percent of devices at home above baseline using smoothed Google mobility data between March and August 2020. (**B**) From the greater Seattle Coronavirus Assessment Network (SCAN), fraction self-reporting they always wear a mask when in public. Data and best fit line with associated 95% confidence interval (CI). (**C**) Estimated R_e_ from epidemiological data (red), modeled reduction in R_e_ due to reduced mobility and masking (solid black), and counterfactual scenario without masks (dashed black). Each line is bounded by 95% CI. Dates marking important events and local policy changes that affected gathering behavior and masking are overlaid.

We next used mobility data from Google^32^ (Fig. 6A), data documenting mask use over time from the greater Seattle Coronavirus Assessment Network (SCAN)^33^ (Fig. 6B) and estimates of the effective reproductive number in King County, Washington state^34^ (Fig. 6C) all through September 2020, to estimate the reduction in R_e_ due to mask wearing. Using a loglinear regression of ${\text{R}}_{{\text{e}}}$ versus mobility, mask usage, and random effects in time, we estimated the reduction in ${\text{R}}_{{\text{e}}}$ due to reduced mobility alone and the additional impact of mask usage. On August 30, 2020, from epidemiological data (i.e. observed COVID-19 cases, hospital admissions, and mortality) alone, we estimate that ${\text{R}}_{{\text{e}}}$ was 0.8 (95% CI [0.5, 1.2]). In a counterfactual scenario with only the observed mobility reduction from the pre-COVID baseline, ${\text{R}}_{{\text{e}}}$ would have been 1.48 (95% CI [1.38, 1.58]). Adding surveyed mask usage to the mobility impact further reduces ${\text{R}}_{{\text{e}}}$ to 1.08 (95% CI [1.03, 1.13]), an absolute reduction of 0.4 and relative reduction of 27%. The residual reveals additional variation not explained by mobility or masking, likely due to unmeasured, focal, high-risk social gathering and workplace behavior (Supplementary Fig. 6). These data are most consistent with the low efficacy (*ε*_*x*_ ~ 0.3–0.6), high coverage scenario for masks described in Fig. 3C, D above.

This inference of relatively low efficacy from a county-level analysis depends on both the physical properties of masks and how they are used. For example, a survey data (*n* = 4273) in Whatcom county, Washington showed that while 92% surveyed reported usually or always wearing masks in public spaces, only ~ 40% did so in “private” situations amongst friends and family who live in separate households^27^. Low mask usage in high-contact settings likely strongly attenuates the average effectiveness of masks at the county-level.

### Predicted impact of antiviral therapy during early symptomatic infection on R_e_

**Figure 7:**
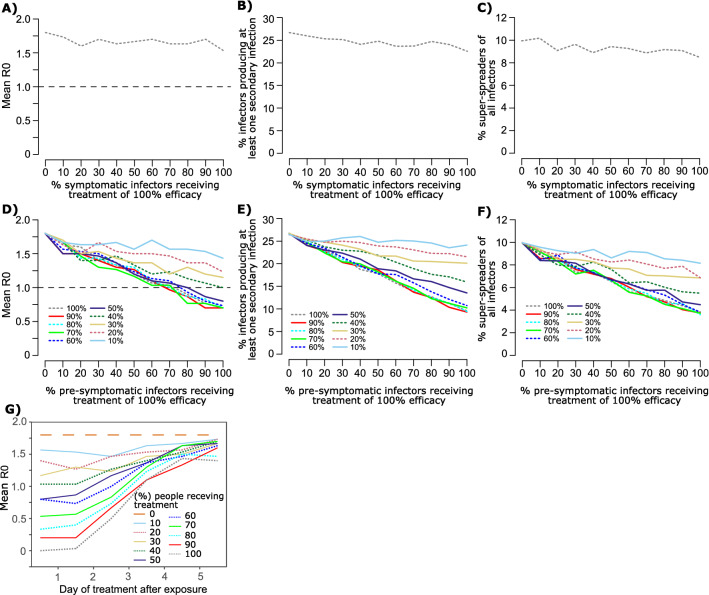
Projected impact of antiviral therapy on SARS-CoV-2 effective reproductive number (R_e_). Each line in each panel is based on simulations of 3000 transmitters with varying daily exposure contacts. Panels (**A**–**C**) assume early symptomatic therapy (before peak viremia, within ~ 5 days after exposure) with 100% efficacy. Panels (**D**–**F**) assume post-exposure prophylaxis given during pre-symptomatic infection with different efficacies (colored lines). (**A** and **D**) Projected R_e_ given different amounts of antiviral uptake in the population, (**B** and **E**) Projected percent of infected people who infect at least one person given different amounts of antiviral uptake in the population. (**C** and **F**) Projected percent of infected people who infect at least 5 people given different amounts of antiviral uptake in the population. (**G**) Projected impact of post-exposure prophylaxis on SARS-CoV-2 reproductive number according to the day of treatment relative to exposure with different percentages of infected individuals receiving antiviral therapy.

Treatment as prevention is a highly effective means for reducing person-to-person transmission of HIV^35,36^. Our models predict that initiation of potent antiviral therapy within ~ 0.5–5 days of the symptoms onset (i.e., early symptomatic therapy) is likely to have therapeutic benefit^37^. We therefore tested whether early symptomatic therapy (before peak viremia, within ~ 5 days after exposure) which rapidly eliminates shedding might also decrease secondary transmissions. Owing to the fact that symptomatic therapy would usually occur after peak viral shedding (Supplementary Fig. 7A), our simulations suggest that even widespread implementation of early symptomatic therapy would not lower R_e_ (Fig. 7A), percentage of infected people who transmit to at least one other person (Fig. 7B) or percentage of infected people who transmit to at least 5 other people (Fig. 7C).

### Predicted impact of post-exposure prophylaxis (PEP) on R_e_

PEP is also a potential method for lowering SARS-CoV-2 transmissions. Because PEP is given in the pre-symptomatic phase and would usually fall before or near peak viral shedding (Supplementary Fig. 7B), our simulations suggest an inverse linear relationship between uptake of PEP and R_e_ (Fig. 7D), percentage of infected people who transmit to at least one other person (Fig. 7E) or percentage of infectors who transmit to at least 5 other people (Fig. 7F). To achieve R_e_ < 1 would require PEP efficacy of 50% and ~ 75% uptake in the population, which would in turn require 75% of SARS-CoV-2 cases to be contact traced. Increases in PEP efficacy beyond 0.5 would provide minimal to no enhancement of this benefit (Fig. 7D).

We further determine that the timing of PEP is critical for success. If PEP is initiated within 2 days of exposure, then the percent of people receiving effective treatment is highly predictive of R_e_ (Fig. 7G). However, from day 3 onwards, the impact of effective PEP diminishes (Fig. 7G).

Overall, these results highlight the fact that masking is likely to have more of an impact on R_e_ than any form of licensed antiviral therapy that emerges during the course of the pandemic.

## Discussion

Relative to other barrier methods for preventing the spread of infectious diseases such as condoms, masks are imperfect^38^. Surgical and cloth masks, which are now used commonly by members of the public, do not completely eliminate droplet and airborne emission of viral particles by a transmitter^1^. Nor do they prevent viral exposure to airway cells among exposed contacts. Their effectiveness in real world settings is further limited by intermittent compliance and improper masking technique. As a result, masks only prevent a proportion of person-to-person transmissions.

Nevertheless, our results demonstrate that in the absence of a licensed vaccine, based on moderate efficacy, low cost, high availability, and ease of use, masks are the most effective currently available biomedical intervention. If implemented widely and strategically, on top of baseline levels of physical distancing, masking would be sufficient to suppress ongoing spread of SARS-CoV-2 until widespread deployment of a vaccine is possible. More specifically, our model suggests that increased masking would lower the effective reproduction number (R_e_), lower the percentage of infected people who transmit the virus, decrease the total number of super-spreader events, and lower the exposure viral loads among infected people, possibly leading to less severe infections overall^29^.

We also used a regression/mixed effects model that incorporates mobility and masking data in Washington state to estimate the reductions in R_e_ due to mask wearing and simulate a counterfactual in which masks were not worn. The results provide a real-world example of our viral load model predictions, suggesting that masking prevented exponential growth of infections for 4 months in Washington state. Without a major increase in mask wearing over the summer, we surmise that a substantial increase in cases and deaths would have occurred and reinstitution of significant physical distancing would have been required.

Importantly, there appears to be a critical threshold of compliance. We predict massive additional benefits accrued with an increase in masking compliance from 75% of people masking 75% of the time to 90% of people masking 90% of the time. Masking also highlights the critical nature of suppressing super-spreader events. If nearly 100% compliance could be achieved among persons with 10 or more exposure contacts per day, then this would be sufficient for maintaining R_e_ less than one. This result highlights that policies mandating the proper use of masks at all times by all persons at sites of known super-spreader events including high risk work environments, locker rooms, weddings, social gatherings, and schools should be considered.

Slight increases in mask efficacy could also drive R_e_ to much lower levels. We believe that different types of masks should be comparatively tested with the same scientific rigor applied to clinical trials of small molecular agents and vaccines.

An important artifact of widespread masking is that while the total number of incident cases is expected to decrease dramatically, the proportion of transmissions in which at least one member of the transmission pair is masked will be higher. Therefore, anecdotal documentation of successful infections between masked individuals, or even super-spreader events in which many infected people were masked, should not be misinterpreted as failure of masking policy. The counterfactual, that masks limited the severity of these events, is likely to be true. Only longitudinal incidence data, along with shifts in level of masking in a given region, are appropriate for inferring the effectiveness of masks.

It is unlikely that antiviral therapy will prove nearly as useful as widespread masking for preventing transmissions. We previously demonstrated that antiviral therapy given during the early symptomatic phase of infection has the potential to limit duration of shedding and infection associated inflammation and is likely to be more efficacious than therapy given later during COVID-19 to hospitalized patients^37^. Unfortunately, early symptomatic therapy would likely occur after peak viral load in a majority of cases. Our simulations suggest that even 100% penetrance of extremely potent antiviral therapies would have a negligible impact on population level spread of the virus. Therefore, while treatment as a prevention is a vital piece of HIV public health policy^36^, it is unlikely to impact the COVID-19 pandemic.

On the other hand, treatment in the earliest pre-symptomatic phase of infection, which could only realistically occur in the setting of PEP, happens prior to peak viral load and therefore could limit secondary transmissions. However, the gains from this approach diminish with each day following exposure. In order to meaningfully impact R_e_, over 50% of exposed contacts would need to receive fully effective therapy within 3 days of an exposure. Given that no available agent yet achieves this level of efficacy and that identifying 50% of post-exposure contacts is unrealistic in most countries, it is clear that relative to masking, PEP will only have an adjunctive role in managing the pandemic. Potential areas of implementation are among high-risk populations such as skilled nursing facility residents or cancer center patients and among populations where masking is difficult or impossible.

Our prior work strongly suggests the presence of a transmission dose response curve in which exposure viral load is a key determinant of transmission risk^39,40^. Our current analysis is built upon this assumption. We project that masks will lead to a lower exposure viral load among newly infected people, particularly if both the transmitter and exposed individual are successfully masked. Animal models of SARS-CoV-1 and MERS^30,31,41^, as well as challenge studies with influenza H1N1 in humans^42^, all demonstrate that lower exposure dose is associated with less severe disease, and human data from the SARS-CoV-1 outbreak in Hong Kong suggest a similar trend^43^. While data for SARS-CoV-2 in humans is lacking, it is notable that age-adjusted hospitalization and death rates may be decreasing since more widespread utilization of masks. A mask related reduction in exposure viral load is a plausible but unproven reason for this observation.

Our work has key limitations. First, based on available data, it is impossible to know the true average efficacy of a mask worn by a transmitter or an exposed contact. Many epidemics at the state level have demonstrated a reduction in the effective reproductive number ranging from 0.2–0.5 when more widespread masking was implemented, even as physical distancing levels waxed and waned. Our model suggests that if 75–90% of people wear masks 75–90% of the time, which is roughly in accordance with state level observations of mask compliance, then a broad estimate for real world mask efficacy is ~ 0.3–0.6, assuming that efficacy is equal between transmitters and exposed contact, and that masks are properly used to optimize their efficacy. If transmitter masking is more efficacious, while exposed contact masking is proportionally less efficacious, then similar results can be expected. The real-world estimate is inclusive of multiple factors including variability in mask type, masking technique and consistency of individual use across different settings. Regardless of the precise estimate, it is clear that wider implementation would yield significant reductions in spread of SARS-CoV-2 at the population level.

Second, our generalized model is not region-specific for the current pandemic. For example, different sampling techniques and PCR assays are employed to measure SARS-CoV-2 viral loads, giving rise to different peak viral loads in different studies, which might affect our estimates of TD50. The relative impact of super-spreader events, intensity of transmission and proportion of symptomatic cases may also vary from region to region based on contact network structure and age demographics. Nevertheless, the general qualitative conclusions about masking are insensitive to these differences and are likely to be generalizable across the globe.

Third, our model does not include a standard SIR format and therefore does not capture other dynamic features that might alter the force of infection such as herd immunity or time-variant shifts in degree of physical distancing. Another missing feature that could be captured with an SIR modeling framework is the possibility of an assortative mixing pattern, in which individuals with lower adherence to masking might preferentially interact with others who have low adherence to masking^12^. Such an effect could allow persistence of SARS-CoV-2 within this sub-population, even if masking is sufficient for containment in the rest of the population.

Fourth, our model assumes that the viral dynamics that is coughed/exteriorized has a similar profile as nasopharyngeal viral loads in a transmitter. The validity of this assumption needs to be verified with carefully designed experiments, which might affect our estimates of TD50. However, the general qualitative conclusions about masking will remain unaffected.

Finally, the low dose inoculum transmitted to the secondary infected person as a result of masking might or might not have an impact on the viral dynamic profile including viral peak in the secondary infected individual. Due to the lack of availability of such data on SARS-CoV-2, we operated under the assumption that viral dynamic profiles are insignificantly altered due to the change in dose inoculum. Hypothetically, if we assume that viral dynamic profiles are significantly changed such that viral peak is decreased due to low inoculum like in the case of influenza^44^, we would expect an even more significant decrease in the effective reproduction number than we projected in this manuscript. Therefore, it is possible that we might be underestimating the impact of the mask use and only projecting the lower bound. In the future, these assumptions can be adequately addressed but will require investigation of the inoculum effect on viral dynamics.

In conclusion, we developed a mechanistic model to demonstrate how masks reduce exposure SARS-CoV-2 viral load and transmission probability. Widespread use of even modestly effective masks is predicted to severely limit epidemic spread and represents the key available intervention along with physical distancing, to mitigate the number of infections, and perhaps the proportion of infections that are severe, while the world awaits a widely available and effective vaccine.

## Materials and methods

### SARS-CoV-2 within-host model

To generate viral loads for transmission, we used the within-host model describing the SARS-CoV-2 infection from our previous study^18^. A detailed description is in the Supplement.

### Dose–response model

We employed our previously developed dose–response model^19^, to estimate the probability of virus entering the airway given a transmitter viral load (i.e., contagiousness) and the probability of cellular infection given a transmitter viral load, (i.e., infectiousness) $P_{t} \left[ {V\left( t \right)} \right]$ (response) based on viral loads $V\left( t \right)$ (dose) as described in the Supplement.

### Transmission model and reproduction number

Our methods for describing number of heterogeneous values for individual effective reproductive number is also previously described^19^, and is detailed in the Supplement. Briefly, based on exposure viral load and number of exposure contacts within a certain time frame, we summed up the number of secondary infections over 30 days since the time of exposure to obtain the individual reproduction number and did this under each parameter scenario for 3000 people. Key parameter values are also in the Supplement^20–23,25^.

### Modeling mask and antiviral use

Reductions in exposure viral load, transmission risk and effective reproductive number based of masking and antivirals are detailed in the Supplement.

### Epidemiological modeling and analysis

Our modeling of COVID-19 epidemiology is described in the Supplement.

## Supplementary Information

Supplementary Information 1.

Supplementary Information 2.
