# Supplementary material for: Slight reduction in SARS-CoV-2 exposure viral load due to masking results in a significant reduction in transmission with widespread implementation
Source: Sci Rep. 2021 Jun 4;11:11838. doi: 10.1038/s41598-021-91338-5 (PMC8178300; doi:10.1038/s41598-021-91338-5)
Supplement: Supplementary file 1 — Supplementary Information 1. [file 41598_2021_91338_MOESM1_ESM.docx]

**Supplementary figure 1. Impact of masking of the transmitter or exposed contact alone or both members of the transmission pair, on transmission risk given an exposure viral load**. Each panel is based on simulations of 1000 transmission pairs. A&B). Absolute reduction in transmission risk. C&D) Relative reduction in transmission risk. A&C) Transmitter is masked only B&D) Both transmitter and exposed contact are masked

**Supplementary figure 2. Impact of transmitter viral load on transmission probability in the context of dual masking**. For varying mask efficacy on exposed and transmitting contacts and increasing viral load of the transmitter (A-D), transmission risk is negligible for lower viral loads of transmitters (<1e7 copies) and medium efficacy masks >0.5. For transmission events at high viral loads, transmission is less likely if the exposed contact wears an effective mask than if the transmitter wears an effective mask.

**Supplementary figure 3. SARS-CoV-2 spread is driven by a very small percentage of the population**. For varying mask efficacy on exposed and transmitting contacts and A&E) Low B&F) moderate C&G) High, and D&H) perfect mask utilization, heatmaps illustrate the percentage of individuals who transmit (A-D) and among individuals who transmit, the percentage who infect >5 others (thus defined as a super spreader).

**Supplementary figure 4. Contribution to basic reproduction number of transmission clusters of varying sizes.** For different mask efficacy, regardless of A) low B) moderate C) High, and D) near-perfect mask utilization, the contribution to R0 is predominantly driven by green/yellow, meaning superspreading events with >5 infected individuals in the transmission cluster.

**Supplementary figure 5. Outsized benefit of masking potential super spreaders.** Simulated average basic reproductive number (R0) from a theoretical scenario in which individuals with >10 exposure contacts per day wear masks 100% of the time. For varying mask efficacy on exposed and transmitting contacts, basic reproductive numbers reach over 1 only in scenarios where masks efficacy for other individuals is very weak, <0.3. Each heat map is based on simulations of 3000 transmitters with varying daily exposure contacts. A) Low B) moderate C) High, and D) perfect mask utilization in other individuals.

**Supplementary figure 6. Multiplicative residual on estimate of effective basic reproductive number R_e_.** Residuals indicate deviations from the model, not explained by reduced mobility or masking. In relation to events in Washington, public mandates (Stay home stay healthy) and (Masks required) may have had secondary impacts on behavior and business closures not measured through mobility and masking along. In particular, Washington had governmental designations that progressed from total lockdown in spring 2020 (Phase 1), through an intermediate (Phase 1.5) and into Phase 2, which for the first time allowed some indoor dining and indoor visiting with other households.

**Supplementary figure 7. Viral load simulations for early and late antiviral therapy.** Five different trajectories for SARS-CoV-2 viral dynamics. Each color used a different parameter set. The same parameter set was simulated with (solid) and without (dashed) therapy. A) Treatment at day 6 or day 7 illustrates delayed post-exposure prophylaxis (PEP). B) Treatment at day 1 or 3 after exposure illustrates early post exposure prophylaxis.
