# Supplementary material for: Slight reduction in SARS-CoV-2 exposure viral load due to masking results in a significant reduction in transmission with widespread implementation
Source: Sci Rep. 2021 Jun 4;11:11838. doi: 10.1038/s41598-021-91338-5 (PMC8178300; doi:10.1038/s41598-021-91338-5)
Supplement: Supplementary file 2 — Supplementary Information 2. [file 41598_2021_91338_MOESM2_ESM.docx]

**Supplementary methods**

***SARS-CoV-2 within-host model.*** We used the within-host model describing the SARS-CoV-2 infection from our previous study (Goyal et al., Sci Adv 2020). This model assumes that the contact of SARS-CoV-2 (*V*) with susceptible cells (*S*) produces infected cells at rate $\beta VS$ which then generates new virus at a per-capita rate $\pi$. The model also incorporates the death of infected cells mediated by (1) the innate responses ($\delta I^{k}$) and (2) the acquired immune responses ($\frac{mE^{r}}{E^{r}+\phi^{r}}$) by SARS-CoV-2-specific effector cells ($E$). The magnitude of the innate immunity is dependent on the infected cell density and the exponent $k$. The nonlinearity of the acquired responses is captured by the Hill coefficient $r$ that allows for rapid saturation of the killing. Finally, the parameter $\phi$ defines level of SARS-CoV-2-specific effector cells at which the killing of infected cells becomes half maximal. In the model, the rise of SARS-CoV-2-specific effector cells rise is described in a two-stage manner. The first stage defines the proliferation of the first precursor cell compartment ($M_{1}$) at rate$\omega IM_{1}$ and differentiation into a second precursor cell compartment ($M_{2}$) at a per capita rate $q$. Finally, second precursor cells differentiate into effector cells at the same per capita rate $q$ and are cleared at rate $\delta_{E}$.

The model is expressed as a system of ordinary differential equations:

$\begin{matrix} \frac{dS}{dt}=-\beta VS \\ \frac{dI}{dt}=\beta VS-\delta I^{k}I-m\frac{E^{r}}{E^{r}+\phi^{r}}I \\ \frac{dV}{dt}= \pi I-\gamma V \\ \frac{dM_{1}}{dt}=\omega IM_{1}-qM_{1} \\ \frac{dM_{2}}{dt}=q(M_{1}-M_{2}) \\ \frac{dE}{dt}=qM_{2}-\delta_{E}E \end{matrix}$ **(1)**

The initial conditions for the model were assumed as $S\left( 0 \right)={10}^{7}$ cells/mL, $I\left( 0 \right)=1$ cells/mL, $V\left( 0 \right)=\frac{\pi I\left( 0 \right)}{c}$ copies/mL, $M_{1}\left( 0 \right)=1$, $M_{2}\left( 0 \right)=0$ and $E_{0}=0$. For simulations we sampled parameter values from a nonlinear mixed-effect model as described in (Goyal et al., 2020b), with the following fixed effects and standard deviation of the random effects (in parenthesis): Log_10_$\beta$: -7.23 (0.2) virions^-1^ day^-1^; $\delta$: 3.13 (0.02) day^-1^ cells^-k^; $k$: 0.08 (0.02); Log10$(\pi$): 2.59 (0.05) day^-1^; $m$: 3.21 (0.33) days^-1^cells^-1^; Log10($\omega$): -4.55 (0.01) days^-1^cells^-1^. We also assumed $r=10$; $\delta_{E}=1$ day^-1^; $q=2.4\times{10}^{-5}$ day^-1^ and $c=15$ day^-1^.

***Dose-response model.*** We employed our previously developed dose-response model to estimate the probability of virus entering the airway given a transmitter viral load (i.e., contagiousness) and the probability of cellular infection given a transmitter viral load, (i.e., infectiousness) $P_{t}[V(t)]$ (response) based on viral loads $V(t)$ (dose) (Goyal et al. eLife 2020). The relation between the response and the dose follows, $P_{t}[V\left( t \right);\lambda,\alpha]=\frac{V\left( t \right)^{\alpha}}{\lambda^{\alpha}+V\left( t \right)^{\alpha}}$ , being $\lambda$ the viral load that corresponds to 50% infectiousness and 50% contagiousness and $\alpha$ the Hill coefficient that controls the sharpness in the dose-response curve. We assumed that the viral load-dependent contagiousness (i.e., the probability that virus is passaged to the exposed person’s airway) is the same as infectiousness. We estimate the transmission risk as the product of the infectiousness and contagiousness (Goyal et al. eLife 2020).

***Transmission model and reproduction number.*** As in our previous model (Goyal et al. eLife 2020), we determined the total exposed contacts of a transmitter within a time step $(\Delta_{t})$ using a gamma distribution, i.e. $\eta_{\Delta_{t}}\sim\Gamma\left( \frac{\theta}{\rho},\rho\right)\Delta_{t}$, where $\theta$ and $\rho$ represent the average daily contact rate and the dispersion parameter, respectively. The true number of exposure contacts (with viral airway exposure) was then obtained by multiplying the total exposed contacts and the contagiousness of the transmitter (i.e., $\zeta_{t}=\eta_{\Delta_{t}}P_{t}$). We modelled infectiousness as a Bernoulli event with mean $P_{t}$, yielding the number of secondary infections within a time step as $T_{\Delta_{t}}=Ber(P_{t})P_{t}\eta_{\Delta_{t}}$. Finally, we summed up the number of secondary infections over 30 days since the time of exposure to obtain the individual reproduction number, i.e.$R_{0}=\sum_{\Delta_{t}} T_{\Delta_{t}}$. For each successful transmission, we further assumed that it takes $\tau$ days for the first infected cell to produce virus.

In simple steps, we followed the procedure below to estimate R_e_,

1. Simulate viral load $V\left( t \right)$ of a simulated infected individual using **the within-host model.**
2. For a given combination of ($\lambda, \tau, \alpha, \theta,\rho$)
   1. For each time step $\Delta_{t}$
      1. Compute $P_{t}\left[ V\left( t \right);\lambda,\alpha\right]$
      2. Draw $\eta_{\Delta_{t}}\sim\Gamma\left( \frac{\theta}{\rho},\rho\right)\Delta_{t}$
      3. Calculate $T_{\Delta_{t}}=Ber\left( P_{t} \right)P_{t}\eta_{\Delta_{t}}$
   2. Calculate $R_{e}=\sum_{\Delta_{t}} T_{\Delta_{t}}$
3. Repeat Steps 1 and 2 to estimate $R_{e}$ for 3,000 infected individuals. The population level $R_{0}$ can then be calculating by taking the mean of 3,000 individual $R_{e}$ values.

***Parameter values for the transmission model.*** For simulations, we used the parameter set [$\alpha$, $\lambda$, $\tau$, $\theta$, $\rho$] = [0.8, 10^7^, 0.5, 4, 40]) as they most closely reproduces empirically observed individual $R_{0}$ and serial interval histograms as well as mean $R_{0}$ across individuals ($R_{0}\in[1.4, 2.5]$) and mean serial interval across individuals (SI $\in[4.0, 4.5]$) early during the pandemic (Adam et al., 2020; Bi et al., 2020; Du et al., 2020; Endo et al., 2020; Zhang et al., 2020).

***Modeling mask use.*** To evaluate the impact of the use of mask on epidemics, we first assumed that a mask decreases the exposure viral load by a fraction $(1-\epsilon)$, being $\epsilon$ the mask efficacy or the proportion of viruses filtered by the mask of transmitter or exposed individuals. If the transmitter is wearing mask with efficacy $\epsilon_{T}$ and the exposed person is wearing a mask with efficacy $\epsilon_{E}$, then the combined mask efficacy $\epsilon_{C}, is given by$ $1-(1-\epsilon_{T})(1-\epsilon_{E})$. Infectiousness or contagiousness reduction by the use of mask can be computed as:

$\xi=1-\frac{P_{t}(\epsilon> 0)}{P_{t}(\epsilon=0)}=\frac{1-\left( 1-\epsilon_{T} \right)^{\alpha}\left( 1-\epsilon_{E} \right)^{\alpha}}{1+\left( 1-\epsilon_{T} \right)^{\alpha}\left( 1-\epsilon_{E} \right)^{\alpha}\frac{V_{t}^{\alpha}}{\lambda^{\alpha}}}$ **(2)**

Similarly, the transmission risk reduction using mask can be computed as:

$\xi^{'}=1-\frac{P_{t}(\epsilon>0)P_{t}(\epsilon>0)}{P_{t}(\epsilon=0)P_{t}(\epsilon=0)}=\xi^{2}+2\xi\frac{\left( 1-\epsilon_{T} \right)^{\alpha}\left( 1-\epsilon_{E} \right)^{\alpha}\left( V_{t}^{\alpha}+\lambda^{\alpha} \right)}{{\left( 1-\epsilon_{T} \right)^{\alpha}\left( 1-\epsilon_{E} \right)^{\alpha}V}_{t}^{\alpha}+\lambda^{\alpha}}.$ **(3)**

Finally, we modeled the *compliance* of an individual wearing mask in the population as a Bernoulli event with mean $\sigma$ and the *adherence* of wearing it at time step $\Delta_{t}$ as a Bernoulli event with mean $\vartheta$. Compliance is defined as whether the person ever wears a mask. Adherence is the percentage of time that a mask wearer wears a mask.

***Simulating secondary transmissions with mask use.*** For a specific scenario with selected $\sigma$ and $\vartheta$, we followed the procedure below to estimate the population level $R_{0}$:

1. Simulate $V\left( t \right)$ for a transmitter using the within-host model in **eq. 1.**
2. Simulate transmitter mask compliance using $Ber(p=\sigma)$.
3. Discretize the time-space of 30 days over time steps $\Delta_{t}$. For each time step,
   1. If the transmitter is *compliant* in using a mask:
      1. Simulate transmitter mask *adherence* at time step $\Delta_{t}$ using $Ber( p= \vartheta)$
      2. If transmitter is wearing a mask at time step $\Delta_{t}$
         1. Draw $\eta_{\Delta_{t}}\sim\Gamma\left( \frac{\theta}{\rho},\rho\right)\Delta_{t}$.
         2. Determine masking adherence among exposed contacts at time step $\Delta_{t}$ using $\kappa\sim Ber(p= \sigma\vartheta)$. If $\kappa=1$, then there is 100% adherence among exposed contacts and if $\kappa=0$, then there is 0% adherence among exposed contacts.
         3. Determine the number of exposed contact wearing a mask (i.e., $\eta_{\Delta_{t\_Mask}}=\eta_{\Delta_{t}}\kappa$) and the number of exposed contact not wearing a mask (i.e., $\eta_{\Delta_{t\_Unmask}}=\eta_{\Delta_{t}}(1-\kappa)$.
            1. Compute $P_{t_{MasktoMask}}\left[ \left( 1-\epsilon_{T} \right)\left( 1-\epsilon_{E} \right)V\left( t \right);\lambda,\alpha\right]$
            2. Calculate $T_{\Delta_{t_{MasktoMask}}}=Ber\left( P_{t_{MasktoMask}} \right)P_{t_{MasktoMask}}\eta_{\Delta_{t_{Mask}}}$
            3. Compute $P_{t_{MasktoUnmask}}\left[ \left( 1-\epsilon_{T} \right)V\left( t \right);\lambda,\alpha\right]$
            4. Calculate $T_{\Delta_{t_{MasktoUnmask}}}=Ber\left( P_{t_{MasktoUnmask}} \right)P_{t_{MasktoUnmask}}\eta_{\Delta_{t\_Unmask}}$
      3. If the transmitter is not adhering with the use of a mask at time step $\Delta_{t}$, which is determined at step (i).
         1. Draw $\eta_{\Delta_{t}}\sim\Gamma\left( \frac{\theta}{\rho},\rho\right)\Delta_{t}$.
         2. Determine masking adherence among exposed contacts at time step $\Delta_{t}$ using $\kappa\sim Ber(p= \sigma\vartheta)$.
         3. Determine the number of exposed contact wearing a mask (i.e., $\eta_{\Delta_{t\_Mask}}=\eta_{\Delta_{t}}\kappa$) and the number of exposed contact not wearing a mask (i.e., $\eta_{\Delta_{t\_Unmask}}=\eta_{\Delta_{t}}(1-\kappa)$
            1. Compute $P_{t_{UnmasktoMask}}\left[ \left( 1-\epsilon_{E} \right)V\left( t \right);\lambda,\alpha\right]$
            2. Calculate $T_{\Delta_{t_{UnmasktoMask}}}=Ber\left( P_{t_{UnmasktoMask}} \right)P_{t_{UnmasktoMask}}\eta_{\Delta_{t_{Mask}}}$
            3. Compute $P_{t_{UnmasktoUnmask}}\left[ V\left( t \right);\lambda,\alpha\right]$
            4. Calculate $T_{\Delta_{t_{UnmasktoUnmask}}}=Ber\left( P_{t_{UnmasktoUnmask}} \right)P_{t_{UnmasktoUnmask}}\eta_{\Delta_{t\_Unmask}}$
   2. If the transmitter is not compliant in wearing a mask
      - 1. Draw $\eta_{\Delta_{t}}\sim\Gamma\left( \frac{\theta}{\rho},\rho\right)\Delta_{t}$.
        2. Determine masking adherence among exposed contacts at time step $\Delta_{t}$ using $\kappa\sim Ber(p= \sigma\vartheta)$.
        3. Determine the number of exposed contact wearing a mask (i.e., $\eta_{\Delta_{t\_Mask}}=\eta_{\Delta_{t}}\kappa$) and the number of exposed contact not wearing a mask (i.e., $\eta_{\Delta_{t\_Unmask}}=\eta_{\Delta_{t}}(1-\kappa)$
           1. Compute $P_{t_{UnmasktoMask}}\left[ \left( 1-\epsilon_{E} \right)V\left( t \right);\lambda,\alpha\right]$
           2. Calculate $T_{\Delta_{t_{UnmasktoMask}}}=Ber\left( P_{t_{UnmasktoMask}} \right)P_{t_{UnmasktoMask}}\eta_{\Delta_{t_{Mask}}}$
           3. Compute $P_{t_{UnmasktoUnmask}}\left[ V\left( t \right);\lambda,\alpha\right]$
           4. Calculate $T_{\Delta_{t_{UnmasktoUnmask}}}=Ber\left( P_{t_{UnmasktoUnmask}} \right)P_{t_{UnmasktoUnmask}}\eta_{\Delta_{t\_Unmask}}$
   3. Calculate $T_{\Delta_{t}}=T_{\Delta_{t_{UnmasktoUnmask}}}+T_{\Delta_{t_{UnmasktoMask}}}+T_{\Delta_{t_{MasktoUnmask}}}+T_{\Delta_{t_{MasktoMask}}}$.
4. Calculate $R_{e}=\sum_{\Delta_{t}} T_{\Delta_{t}}$.
5. Repeat Steps **1** to **4** to estimate $R_{0}$ for 3,000 infected individuals (transmitters). R_e_ can then be calculating by taking the mean of 3,000 individual $R_{0}$ values.

***Epidemiological modeling and analysis***. We estimated the time varying effective reproductive number $R_{e}$ in King County, Washington from daily case, hospitalization, and death data provided under a data use agreement by the Washington State Department of Health as described elsewhere (https://covid.idmod.org/data/One_state_many_outbreaks.pdf). We then performed a regression analysis to estimate the contributions to the effective reproductive number $R_{e}$ over time of mobility ($f_{h}$, the fraction staying at home all day above baseline), mask wearing ($f_{M}$, the fraction who report always wearing a mask when outside the home), the estimated fraction of the population not yet infected (susceptible fraction), and a time varying IID random effect to capture unexplained variation $\zeta$.

$\ln\left( R_{e} \right)\sim\ln(1-f_{h})+\ln\left( 1-f_{M} \right)+A\left( \ln S \right)+\zeta$ (4)

The regression model is then used to estimate what $R_{e}$ would be under the observed mobility trends but in the absence of masks ($f_{M}$=0), and that is compared to the estimate with both mobility trends and mask usage. Mask survey data, mobility changes, and the negligible impact of declining susceptibility do not completely capture the variation in $R_{e}$ summarized by the random effects which may be due to specific workplace and social gathering behavior not represented by these county-scale metrics.

For our measure of overall mobility changes, we use the “percent at home above baseline” metric from Google (https://www.google.com/covid19/mobility), and results were largely insensitive to the mobility metric chosen among options from multiple providers. The fraction susceptible is estimated alongside $R_{e}$ and has a negligible impact on this analysis with >90% of the King County population estimated to be as yet uninfected at the time of the analysis. For mask usage over time, we used the daily self-selected survey data from the greater Seattle Coronavirus Assessment Network (SCAN) (https://publichealthinsider.com/wp-content/uploads/2020/08/8.3-SCAN-Technical-Report-3_FINAL_CLEAN.pdf). This data is uniquely useful because the survey has been running daily since May 7, and so catches trends not available in any other surveys known to us. However, the self-selected population is at slightly lower risk than a representative sample from the region. For the mask survey question, we find that the SCAN “always mask outside the home” corresponds closely to the Delphi COVIDCast weighted representative survey “sometimes/always mask” response in the month of overlap (https://delphi.cmu.edu/blog/2020/10/12/new-and-improved-covid-symptom-survey-tracks-testing-and-mask-wearing/)). To estimate mask coverage prior to the survey start on May 7, we assume linear interpolation in the logit space from 1% coverage on March 1 (estimated by Famulare).

***Modeling antiviral treatment***. We simulate the antiviral treatment by assuming that the antiviral treatment reduces the viral production ($\pi$) by ($1-\epsilon_{treat}$), where $\epsilon_{treat}$ is the efficacy of treatment. Here, $\epsilon_{treat}=0$ and $\epsilon_{treat}=1$ represent the case of completely ineffective and 100% effective treatment, respectively. For the transmission simulations we model *coverage* to treatment with as a Bernoulli event with mean $\psi$.

In the presence of treatment with mean coverage $\psi$ and efficacy $\epsilon_{treat}$, we follow the procedure below to estimate the population level $R_{e}$:

1. Determine adherence to treatment using $Ber( p=\psi)$
2. Determine the time of start of antiviral treatment ($T_{treat}$) for treatment in symptomatic phase and pre-symptomatic phase by randomly drawing a number from Uniform distributions ($U(0.5+I_{I},5+ I_{I})$) and ($U(0.5,5)$), respectively, where $I_{I}$ is the incubation period of the infected individual.
3. Simulate viral load $V\left( t \right)$ of a simulated infected individual using the within-host model in  **eq. 1** with $\epsilon_{treat}=0$ for $t\leq T_{treat}$ and ${0<\epsilon}_{treat}\leq1$ for $t>T_{treat}$**.**
4. For a given combination of ($\lambda, \tau, \alpha, \theta,\rho$)
   1. For each time step $\Delta_{t}$
      1. Compute $P_{t}\left[ V\left( t \right);\lambda,\alpha\right]$
      2. Draw $\eta_{\Delta_{t}}\sim\Gamma\left( \frac{\theta}{\rho},\rho\right)\Delta_{t}$
      3. Calculate $T_{\Delta_{t}}=Ber\left( P_{t} \right)P_{t}\eta_{\Delta_{t}}$
   2. Calculate $R_{e}=\sum_{\Delta_{t}} T_{\Delta_{t}}$
5. Repeat Steps 1 and 4 to estimate $R_{e}$ for 3,000 infected individuals. The population level $R_{e}$ can then be calculating by taking the mean of 3,000 individual $R_{e}$ values.

**References**

Adam, D., Wong, J., Lau, E., Tsang, T., Cauchemez, S., Leung, G., and Cowling, B. (2020). Clustering and superspreading potential of severe acute respiratory syndrome coronavirus 2 (SARS-CoV-2) infections in Hong Kong. Europe PMC *10.21203/rs.3.rs-29548/v1*.

Bi, Q., Wu, Y., Mei, S., Ye, C., Zou, X., Zhang, Z., Liu, X., Wei, L., Truelove, S.A., Zhang, T.*, et al.* (2020). Epidemiology and transmission of COVID-19 in 391 cases and 1286 of their close contacts in Shenzhen, China: a retrospective cohort study. Lancet Infect Dis *20*, 911-919.

Du, Z., Xu, X., Wu, Y., Wang, L., Cowling, B.J., and Meyers, L.A. (2020). Serial Interval of COVID-19 among Publicly Reported Confirmed Cases. Emerg Infect Dis *26*.

Endo, A., Abbott, S., Kucharski, A.J., Funk, S., and Group, C.f.t.M.M.o.I.D.C.-W. (2020). Estimating the overdispersion in COVID-19 transmission using outbreak sizes outside China. Wellcome Open Res *5*, 67.

Goyal, A., Cardozo-Ojeda, E.F., and Schiffer, J.T. (2020a). Potency and timing of antiviral therapy as determinants of duration of SARS CoV-2 shedding and intensity of inflammatory response. medRxiv, 2020.2004.2010.20061325.

Goyal, A., Reeves, D.B., Cardozo-Ojeda, E.F., Schiffer, J.T., and Mayer, B.T. (2020b). Wrong person, place and time: viral load and contact network structure predict SARS-CoV-2 transmission and super-spreading events. medRxiv, 2020.2008.2007.20169920.

<https://covid.idmod.org/data/One_state_many_outbreaks.pdf>. Thakkar, N and Famulare, M. One state, many outbreaks: a transmission modeling perspective on current COVID-19 trends in King, Pierce, and Yakima counties (2020). .

<https://delphi.cmu.edu/blog/2020/10/12/new-and-improved-covid-symptom-survey-tracks-testing-and-mask-wearing/>).

<https://publichealthinsider.com/wp-content/uploads/2020/08/8.3-SCAN-Technical-Report-3_FINAL_CLEAN.pdf>. Roy Burstein et al.  SCAN Data Results and Technical Report #3 (2020)

<https://www.google.com/covid19/mobility>. *Google COVID-19 Community Mobility Reports*.

Zhang, Y., Li, Y., Wang, L., Li, M., and Zhou, X. (2020). Evaluating Transmission Heterogeneity and Super-Spreading Event of COVID-19 in a Metropolis of China. Int J Environ Res Public Health *17*.
